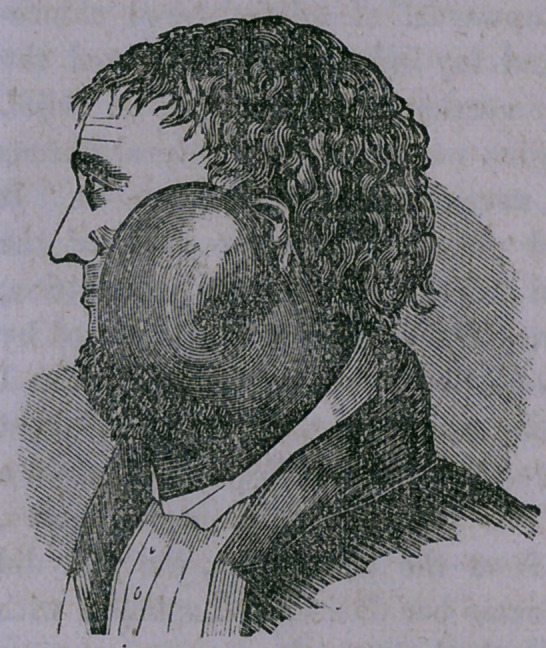# A Fibrous Tumor of the Right Side of the Face

**Published:** 1866-06

**Authors:** 


### CLINICAL REPORTS.

### RUSH MEDICAL COLLEGE DISPENSARY—SURGICAL CLINIC BY DR. E. POWELL.

*A Fibrous Tumor of the Right Side of the Face, involving the Parotid Gland and Common Carotid Artery, of Twenty-one Years' Growth—Removal—Death on the Fourth Day from an Accidental Hemorrhage.*

Giles Hutchinson, of Illinois, about 41 years of age, came to the College Clinic, January 15th, 1866, for the purpose of having removed an immense tumor of the right parotid region.

As imperfectly shown in the cut, it elevated the ear more than an inch, extending from the zygoma downwards over the sheath of the great vessels to within an inch and a half of the clavicle, extending anteriorly considerably in front of masseter muscle, and posteriorly as far as the transverse processes of the upper cervical vertebræ.

The circumference was eighteen inches; its diameters six and eight inches. This large mass was firm, whilst its short projecting lobes were as unyielding as bone.

The steady growth, the impediment to the motions of the jaws and throat, as well as the pain experienced in the part, determined the patient upon the removal of the tumor as the only relief from a speedy and otherwise inevitable death.

Assisted by Drs. Lackey, Durham, and several other physicians, the operation was performed before the class. It was commenced by a vertical incision from a point a little in front of the ear to an inch below its lower border. The integument was then stripped from its surface with the fingers, and the sterno-mastoid, which was inserted into and firmly bound down in its lower portion, was torn across. At this stage of the operation, a ligature was placed upon the common carotid artery, when, by strong and repeated efforts, the tumor was wrenched from its bed. There was very little hemorrhage from the recurrent circulation. During the operation two other small vessels besides the main trunks were tied.

The cavity was large, showing completely the walls of the space occupied by the parotid gland and the styloid process, while it extended down nearly to the clavicle. After the first shock of the operation had subsided, the patient experienced little constitutional irritation, suffering chiefly from tenderness of the pharynx whilst swallowing.

January 19th. The case had progressed extremely well up to this time, when, after rather a full meal, the patient was taken with vomiting, during which the artery was ruptured, and a profuse hemorrhage took place. A ligature was re-applied, but the patient died in twelve hours, exhausted by the loss of blood.

## Figures and Tables

**Figure f1:**